# Long non-coding RNA SNHG5 promotes glioma progression via miR-205/E2F3 axis

**DOI:** 10.1042/BSR20190668

**Published:** 2019-07-19

**Authors:** Xiaojian Li, Liang Liu, Yidan Luo, Sitong Cui, Wei Chen, Ailiang Zeng, Yan Shi, Liangsheng Luo

**Affiliations:** 1 Department of Neurosurgery, Nanjing First Hospital, Nanjing Medical University, Nanjing 210006, China; 2 School of Pharmacy, Nanjing Medical University, Nanjing 211166, China; 3 Department of Neurosurgery, The First Affiliated Hospital of Nanjing Medical University, Nanjing 210029, China; 4 Department of Neurology, Brigham and Women’s Hospital and Harvard Medical School, Boston 02115, MA, U.S.A.

**Keywords:** ceRNA, E2F3, glioma, large intervening non-coding RNA, SNHG5

## Abstract

In recent years, many studies have reported on the abnormal expression and correlation of long non-coding RNAs (lncRNAs) in tumours. However, the accurate molecular mechanism of lncRNAs in glioma is still in its infancy. In the present study, we aimed to explore the molecular mechanism of small nucleolar RNA host gene 5 (SNHG5) in glioma progression. First, we found that SNHG5 expression was higher in glioma and was related to glioma glucose uptake, migration and invasion. Second, through a series of assays, we concluded that SNHG5 acts as a sponge for miR-205, which inhibits tumour growth in glioma by targeting E2F transcription factor 3 (E2F3). Third, using a xenograft mouse model, we demonstrated that SNHG5 regulates tumourigenesis *in vivo*. Taken together, our results show that the SNHG5/miR-205/E2F3 axis is involved in glioma progression and may provide a new therapeutic target for the diagnosis and therapy of glioma.

## Introduction

Glioma accounts for 70% of adult primary pernicious brain tumours [1–3]. Glioblastoma multiforme (GBM) is the most aggressive and malignant type of glioma, with a poor prognosis and high mortality due to the highly proliferative and invasive nature of the tumour [4,5]. Hence, it is critical to expound the molecular mechanisms underlying glioma progression and design reasonable therapeutic interventions for the early diagnosis and treatment of this disease.

Long non-coding RNAs (lncRNAs), a set of non-coding RNAs (ncRNAs) with a length of more than 200 nucleotides, make a significant contribution in cancer biology and serve as biomarkers and therapeutic targets for many human neoplastic diseases [6–8]. In recent years, the aberrant expression of lncRNAs has been discovered in various types of tumours, acting as oncogenes or tumour suppressors [9–11]. Two molecular mechanisms of lncRNAs in the development of glioma have been proposed. One theory suggests that lncRNAs regulate downstream signalling pathways by binding to specific proteins, while the other suggests that lncRNAs regulate downstream signalling pathways by binding to microRNAs (miRNAs) [12]. Comprehending the role of cancer-related lncRNAs to uncover new insights about the mechanisms of tumour progression is of far-reaching significance.

Small nucleolar RNA host gene 5 (SNHG5), a cytoplasmic lncRNA, also known as U50HG, is 524 bp in length. SNHG5 consists of six exons and two nucleolar RNAs (snoRNAs) on human chromosome 6q14.3 [13]. The abnormal expression of SNHG5 has been found in many human cancers, such as bladder cancer, gastric cancer and colorectal cancer [14–16]. However, there are no reports on the molecular mechanism of SNHG5 in glioma. Our study found an increase in the expression of SNHG5 in glioma and that SNHG5 was closely related to glioma glucose uptake, migration and invasion. Next, through a series of assays, we concluded that SNHG5 functions as a sponge for miR-205, which inhibits tumour growth in glioma by targeting E2F transcription factor 3 (E2F3). Third, using a xenograft mouse model, we demonstrated that SNHG5 regulates tumourigenesis *in vivo*. Taken together, these results suggest that SNHG5 may be a potential prognostic factor and therapeutic target for glioma.

## Materials and methods

### Human tissues

The analysis of SNHG5 expression from TCGA database was performed at the GEPIA website (http://gepia.cancer-pku.cn/index.html). Human glioma tissues and normal tissues were obtained from glioma patients who were treated with tumour resection (from June 2010 to April 2016) and brain trauma patients who were treated with internal decompression (from July 2010 to April 2016) surgery at Nanjing First Hospital, Nanjing Medical University, respectively. The present study was approved by the Human Research Ethics Committee of Nanjing First Hospital.

### Cell lines and cell culture

The human glioma cell lines U87 and U251 were purchased from the Chinese Academy of Science Cell Bank (Shanghai, China), and both were maintained in Dulbecco’s modified Eagle’s medium (DMEM, Gibco, NY, U.S.A.) supplemented with 10% fetal bovine serum (FBS, ScienCell, LA, U.S.A.) and antibiotics (100 U/ml penicillin and 100 μg/ml streptomycin). Normal human astrocytes (NHAs) were purchased from JENNIO Biological Technology (Guangzhou, China). All cell lines were maintained in a 37°C, 5% CO_2_ incubator.

### Plasmid constructs, Oligonucleotides and cell transfection

Oligonucleotides were chemically synthesized by GenePharma (Shanghai, China), and the sequences were as follows: SNHG5-small interfering RNA (si-SNHG5) sense 5′- CAG UGA AGA UAA UGA AUG UTT -3′, antisense 3′-ACA UUC AUU AUC UUC ACU GTT-5′; shRNA used to target SNHG5 (sh-SNHG5) sense 5′-CAG UGA AGA UAA UGA AUG UTT-3′, antisense 3′-ACA UUC AUU AUC UUC ACU GTT-5′; miR-205 mimic: 5′-GAT TTC AGT GGA GTG AAG TTC AGG AGG CAT-3′; miR-205 inhibitor: 5′-CAG ACU CCG GUG GAA UGA AG GA-3′; and E2F3-small interfering RNA (si-E2F3): 5′- GAC UUC AUG UGU AGU UGA UU-3′. Lipofectamine 2000 reagents (Invitrogen, Carlsbad, CA, U.S.A.) were used for cell transfection following manufacturer’s protocols.

### Real-time quantitative reverse transcription PCR

Total RNA was isolated with TRIzol (Invitrogen, Carlsbad, CA, U.S.A.) according to the manufacturer’s protocol. cDNA was obtained by using Fermentas reverse transcription reagents and quantitative reverse transcription PCR (qRT-PCR) was conducted with SYBR Green PCR Master Mix (Applied Biosystems, Thermo Fisher Scientific, MA, U.S.A.) following manufacturer’s protocols. The primer sequences used in the present study are as follows: SNHG5 forward 5′-CGA GTA GCC AGT GAA GAT AATG-3′; SNHG5 reverse 5′-CAC ACA ACA GTC AAG TAA ACC-3; miR-205 forward 5′-AAT CCT TCA TTC CAC CGG-3′; miR-205 reverse 5′-GTG CAG GGT CCG AGGT-3′; E2F3 forward 5′-CAC TTC CAC CAC CTC CTG TT-3′; E2F3 reverse 5′-TGA CCG CTT TCT CCT AGC TC-3′; GAPDH forward 5′- GTC AAC GGA TTT GGT CTG TATT-3′; GAPDH reverse 5′- AGT CTT CTG GGT GGC AGT GAT-3′. Relative expression was calculated using the 2^−ΔΔ*C*^_t_ method.

### Western blot analysis

Total protein was extracted from glioma cells using RIPA buffer (KenGen, China) and the concentration of extracted protein was detected by the bicinchoninic acid (BCA) protein assay kit (Thermo Fisher Scientific, MA, U.S.A.). Subsequently, extracted protein samples were separated by sodium dodecyl sulfate polyacrylamide gel electrophoresis and transferred to PVDF membranes (Immobilon, MA, U.S.A.). The membranes were blocked in 5% non-fat milk and incubated with primary antibodies against E2F3 (1:1000, Abcam, CA, U.S.A.) overnight. After washing, the membranes were incubated with HRP-conjugated goat anti-rabbit secondary antibodies (1:2000 dilution, YI FEI XUE BIO TECH, China). GAPDH (1:1000 dilution, YI FEI XUE BIO TECH, China) was used as a control.

### Glucose uptake assay

Glucose uptake was determined using a 2-Deoxyglucose Glucose Uptake Assay Kit (Abcam, CA, U.S.A.) according to the manufacturer’s protocol. Briefly, U87 and U251 cells were cultured in 96-well plates (1000 cells/well) overnight. After 24 h, the cells were incubated in the dark with 2-deoxyglucose (10 mM) for 20 min at 37°C in CO_2_ humidified atmosphere and subjected to the measurement of 2-deoxyglucose uptake using fluorescence micro-plate reader at Ex/Em = 535/587 nm) [17].

### Migration and transwell assays

The migration and transwell assays were performed to assess cell migration and invasion ability. For the invasion assay, the chamber inserts (Merck Millipore, Germany) were pre-coated with 45 μl of Matrigel (1:8 dilution; BD Bioscience, U.S.A.). For both assays, 5 × 10^4^ cells in serum-free medium were seeded into the top chambers, and DMEM containing 10% FBS was added to the lower chamber. After incubation for 24 h at 37°C in a 5% CO_2_ incubator, the chambers were fixed using 4% paraformaldehyde (YI FEI XUE BIO TECH, China), stained with crystal violet solution (YI FEI XUE BIO TECH, China) for 30 min, and washed three times with PBS (Gibco, NY, U.S.A.). Stained cells were observed under an optical microscope and counted and the mean was calculated.

### Luciferase reporter assay

The binding sites of miR-205 on SNHG5 and E2F3 were predicted through bioinformatics websites. The 3′-UTR fragments of SNHG5 and E2F3 interacting with miR-205 were constructed into pMIR-REPORT vectors. Then U251 and U87 cells were co-transfected with the hsa-miR-205 mimic or related miRNAs and reporter constructs. Subsequently, the luciferase activity was measured using a Dual Luciferase Reporter Assay System (Promega, WI, U.S.A.) according to the manufacturer’s instructions.

### MS2-RIP assay

The MS2-RIP assay was carried out to determine whether miR-205 is associated with SNHG5. U87 and U251 cells were both transfected with pcDNA-SNHG5-MS2, pcDNA-SNHG5-mut-MS2 or pcDNA-MS2 for 48 h. Subsequently, the cells were used to conduct RIP using a GFP antibody (Roach) and the Magna RIPTM RNA-Binding Protein Immunoprecipitation Kit (Millipore, U.S.A.) according to the manufacturer’s protocol [18].

### RNA pull-down assay

An RNA pull-down assay was performed to determine the interaction between the lncRNA SNHG5 and miR-205 in glioma. Briefly, the miR-205, miR-205-mut and negative control (NC) probes were synthesized and biotinylated by GenePharma (Shanghai, China). The RNA pull-down assay was conducted using the Magnetic RNA-Protein Pull-Down Kit (Thermo Fisher, NY, U.S.A.) according to the manufacturer’s protocol. U87 and U251 cells were transfected with biotinylated miRNA. Then M-280 streptavidin magnetic beads (Invitrogen, Carlsbad, CA, U.S.A.) were used to incubate cell lysates. Finally, the lncRNA SNHG5 levels was determined by qRT-PCR [19].

### Immunofluorescence

Cells seeded on a 96-well plate, fixed in 4% formaldehyde solution and permeabilized with 0.05% Triton X-100 for 30 min. Next, cells were blocked with 5% goat serum for 1 h and incubated with an E2F3 primary antibody (1:1000 dilution, Abcam, CA, U.S.A.) at 4°C for 12 h, followed by incubation with a FITC-conjugated secondary antibody (1:1000 dilution, CST, MA, U.S.A.) for 1 h. Then, cells were stained with DAPI. Finally, images were taken under fluorescence microscope (OLYMPUS, Tokyo, Japan).

### Xenograft tumour assay

Ten immunodeficient male nude mice (Beijing Laboratory Animal Center, Beijing, China) aged 5–6 weeks were maintained under specific pathogen-free (SPF) conditions. These nude mice were randomly assigned into two groups (five mice per group). A total of 1.0 × 10^7^ U87 cells stably expressing NC or sh-SNHG5 were subcutaneously injected into the mice. After 30 days, the nude mice were killed, and the tumour tissues were stripped and weighed. The expression of SNHG5, miR-205 and E2F3 was measured by PCR or/and Western blot.

### Statistical analysis

All data are expressed as the mean ± standard error, and SPSS 13.0 software was used for data statistical analysis. A *t* test (two groups) or one-way ANOVA (no less than three groups) was used to analyse the statistical significance. Differences of *P*<0.05 (*) were considered statistically significant, and *P*<0.01 (**) was considered statistically very significant. All experiments were repeated three times independently.

## Results

### SNHG5 expression is up-regulated in glioma tissues and glioma cell lines

**Figure 1:**
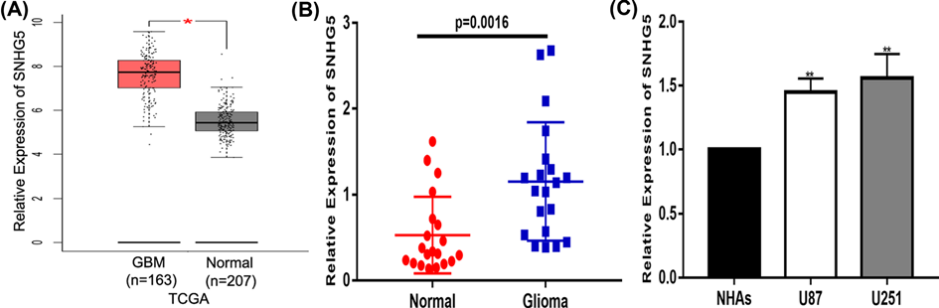
SNHG5 expression is up-regulated in glioma tissues and cell lines (**A**) Expression patterns of SNHG5 in the TCGA database. (**B**). Expression of SNHG5 in clinic glioma tissues. (**C**). Relative SNHG5 expression levels in glioma cell lines (U87 and U251) and NHAs. *P<0.05, **P<0.01.

First, we analysed the expression profiles of SNHG5 in the TCGA database and found that the expression level of SNHG5 in glioma tissues was significantly higher than that in non-malignant tissues (Figure 1A). Next, we detected the expression of SNHG5 in glioma tissues (*n*=20) and normal brain tissues (*n*=20). The results indicated that compared with normal tissues, the expression level of SNHG5 is significantly increased in gliomas (Figure 1B). Additionally, we detected a significant increase in SNHG5 expression in glioma cell lines (U87 and U251) compared with that in NHAs (Figure 1C). These data illustrate that SNHG5 may play a pivotal role in promoting the malignant evolution of glioma.

### SNHG5 promotes glioma cell glucose uptake, migration and invasion

**Figure 2:**
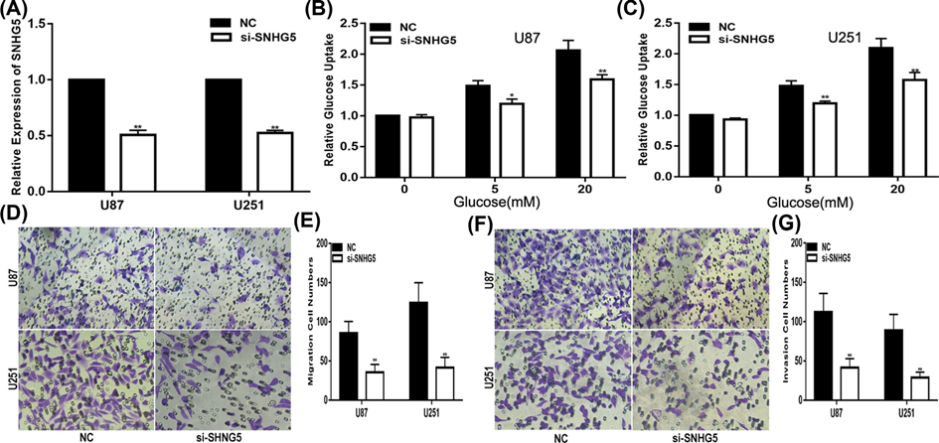
SNHG5 promotes glioma glucose uptake, migration and invasion (**A**) Relative expression level of SNHG5 in U87 and U251 cells transfected with NC or si-SNHG5. (**B,C**). Glucose uptake assay was used to measure the glucose uptake of cells transfected with NC or si-SNHG5. (**D,E**). Migration assay was performed to explore the migration capacity of cells transfected with NC or si-SNHG5. (**F,G**). Transwell assay was applied to explore the invasion ability of cells transfected with NC or si-SNHG5. *P<0.05, **P<0.01.

To investigate the effect of SNHG5 on glioma cells, the expression of SNHG5 was decreased by si-SNHG5 in U87 and U251 cells. First, we confirmed the transfection efficiency in these cell lines by qRT-PCR (Figure 2A). Studies have shown that glucose metabolism has drawn a significant amount of attention in cancer research. We wondered whether SNHG5 could affect glucose metabolism in glioma [20]. Thus, we performed a glucose uptake assay, and the results manifested that compared with the NC, the down-regulation of SNHG5 significantly decreased the ability of cell lines to uptake glucose (Figure 2B,C). Moreover, compared with the NC group, the migration ability of si-SNHG5 U87 and U251 cells was impaired as well (Figure 2D,E). A similar result was obtained in the transwell assay (Figure. 2F,G). These results suggest that SNHG5 promotes glucose uptake, migration and invasion in glioma cell lines.

### SNHG5 sponges miR-205 which suppresses glioma glucose uptake, migration and invasion

**Figure 3:**
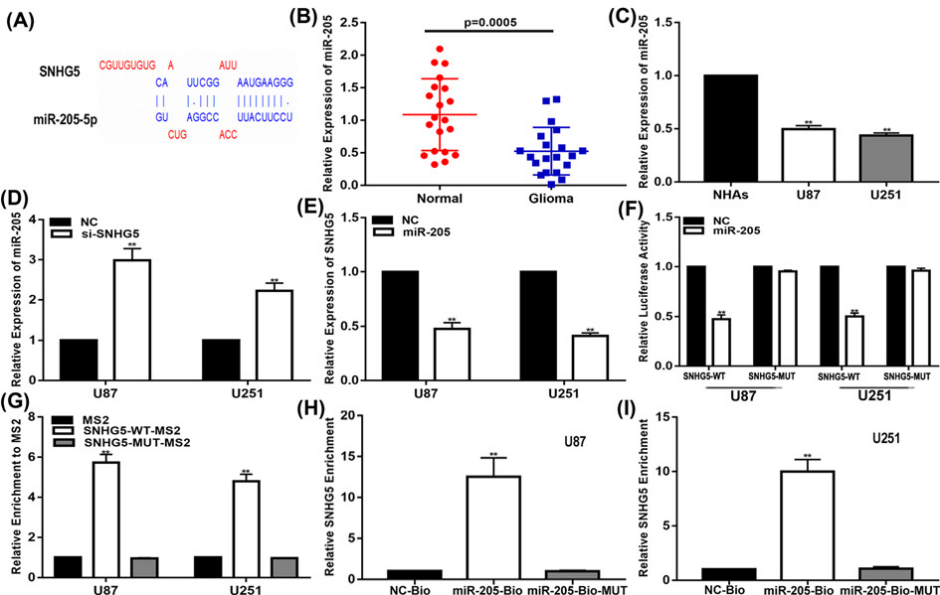
SNHG5 sponged miR-205 in glioma cells (**A**) StarBase v3.0 was used to predict the putative-binding sites of miR-205 on the SNHG5 transcript. (**B**) Expression of miR-205 in clinic glioma tissues. (**C**) Expression of miR-205 in glioma cell lines (U87 and U251) and NHAs. (**D**) Expression of miR-205 in glioma cells transfected with NC or si-SNHG5. (**E**) Expression of SNHG5 in glioma cells transfected with NC or miR-205. (**F**) Luciferase reporter assay showed that miR-205 decreased the luciferase activity of SNHG5 luciferase reporters obviously. (**G**) MS2-RIP was performed to detect endogenous miR-205 associated with the MS2-tagged SNHG5 transcript. (**H,I**) Biotin-based pull-down assay was applied to analyse glioma cells transfected with biotin-labelled miR-205, mutated oligos or biotinylated NC. **P<0.01.

Accumulating evidence has shown that lncRNAs can act as competing endogenous RNA (ceRNAs) during tumourigenesis. CeRNAs can interact with functional miRNAs to regulate gene expression. Through the online database starBase v3.0 (http://starbase.sysu.edu.cn/), we found that miR-205 may be a target of SNHG5 (Figure 3A). To determine whether SNHG5 can interact with miR-205 in glioma, we performed the following experiments. First, we explored the expression of miR-205 in glioma tissues and cell lines. The results showed that miR-205 was down-regulated in glioma tissues and cell lines compared with that in normal brain tissues and NHAs (Figure 3B,C). Second, through qRT-PCR, we determined that miR-205 expression levels were up-regulated after down-regulating SNHG5 in glioma cells and that miR-205 mimics could also down-regulate SNHG5 expression levels in glioma cells (Figure 3D,E). In addition, SNHG5 luciferase reporter plasmids with wild-type and predicted mutant sites for miR-205 were constructed. We found that miR-205 mimics decreased the luciferase activity of the wild-type plasmid but did not reduce the luciferase activity of the mutant plasmid (Figure 3F). Furthermore, MS2-RIP was conducted to verify the binding interaction between miR-205 and SNHG5. The results showed that, compared with the empty vector and MS2-tagged mutant-type SNHG5, MS2-tagged wild-type SNHG5 was enriched for miR-205 (Figure 3G). In addition, we carried out an RNA pull-down assay, and the results illustrated that SNHG5 was pulled down by the target oligos (Figure 3H,I). These findings clarify that SNHG5 is a sponge for miR-205.

**Figure 4:**
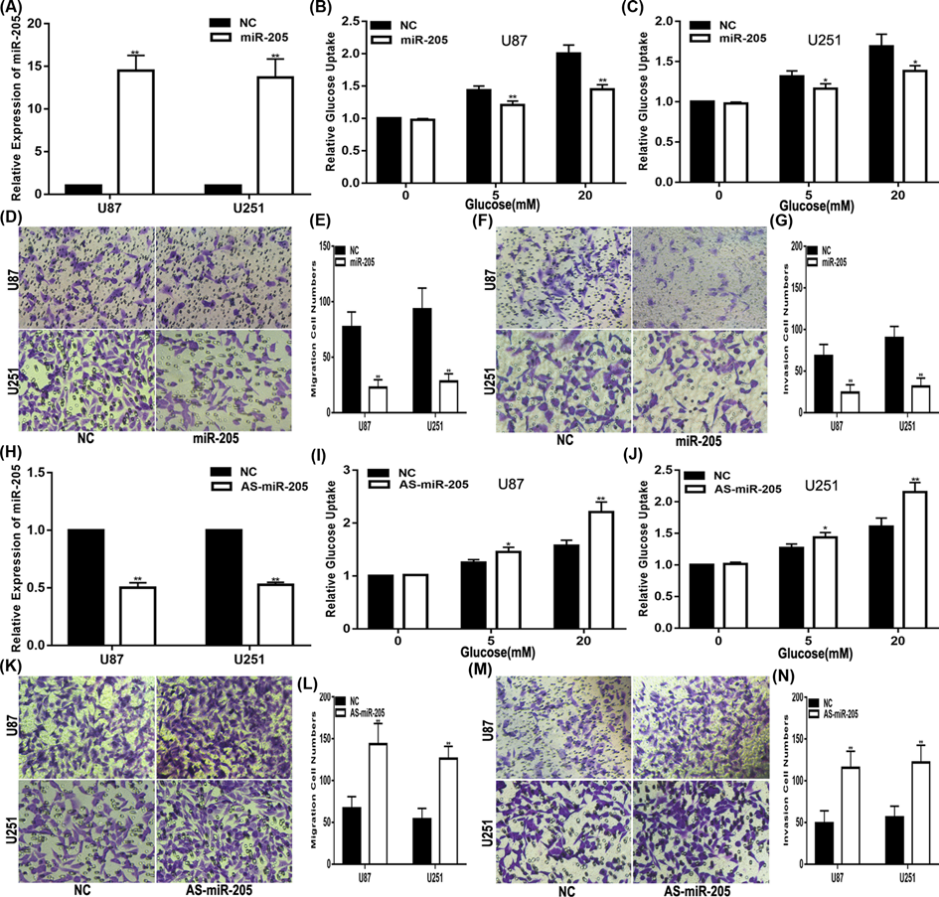
The effect of miR-205 on glioma cell lines (**A**). Relative expression level of miR-205 in U87 and U251 cells transfected with NC or miR-205 mimic. (**B,C**). Glucose uptake assay was used to measure the glucose uptake of cells transfected with NC or miR-205 mimic. (**D,E**). Migration assay was performed to explore the migration capacity of cells transfected with NC or miR-205 mimic. (**F,G**). Transwell assay was applied to explore the invasion ability of cells transfected with NC or miR-205 mimic. (**H**). Relative expression level of miR-205 in U87 and U251 cells transfected with NC or miR-205 inhibitor. (**I,J**). Glucose uptake assay was used to measure the glucose uptake of cells transfected with transfected with NC or miR-205 inhibitor. (**K,L**). Migration assay was performed to explore the migration capacity of cells transfected with transfected with NC or miR-205 inhibitor. (**M,N**). Transwell assay was applied to explore the invasion ability of cells transfected with NC or miR-205 inhibitor. *P<0.05, **P<0.01.

To expound the biological function of miR-205 in glioma, miR-205 mimics or inhibitors were transfected into glioma cell lines. Then, the transfection efficiency was confirmed by qRT-PCR (Figure 4A–H). Through these experiments mentioned above, we determined that the glucose uptake of both glioma cell lines was decreased/increased with the up-regulation/down-regulation of miR-205, respectively (Figure 4B,C,I,J). Additionally, the migration and invasion abilities were enhanced or suppressed by treatment with the miR-205 inhibitor or the miR-205 mimic, respectively (Figure 4D–G,K–N). In conclusion, our data strongly suggest that SNHG5 exerts its biological effects via sponging endogenous miR-205.

### miR-205 regulates glioma glucose uptake, migration and invasion via targeting E2F3

**Figure 5:**
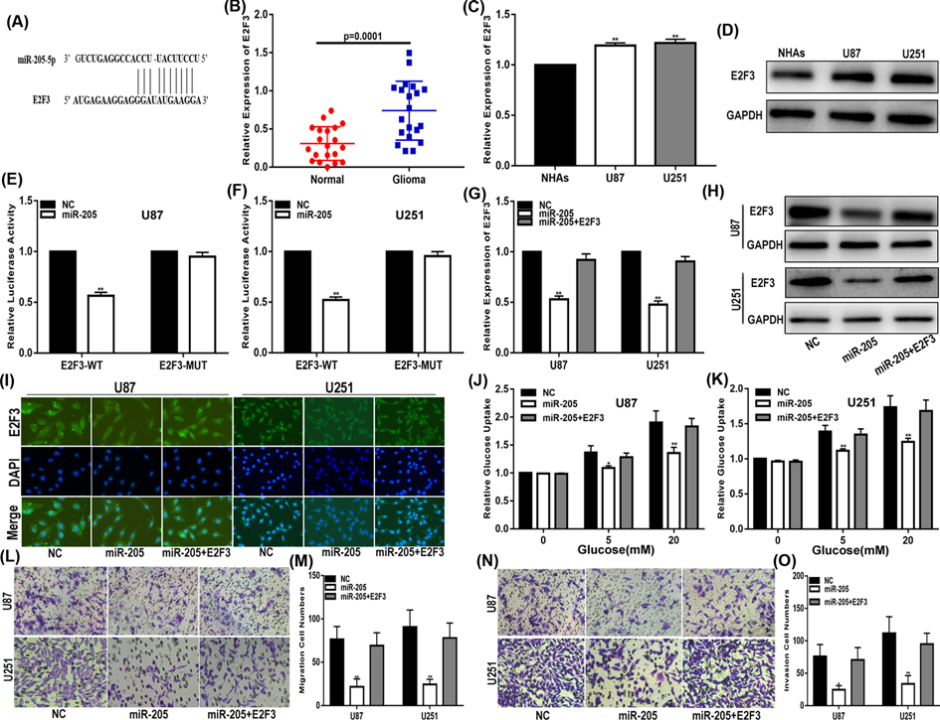
MiR-205 regulates glioma glucose uptake, migration and invasion by targeting E2F3 (**A**) StarBase v3.0 was used to predict the binding site of miR-205 within the 3′-UTR of E2F3. (**B**) Expression of E2F3 in clinic glioma tissues. (**C,D**) Expression of E2F3 in glioma cell lines and NHAs (qRT-PCR and Western blot). (**E**-**F**) Luciferase reporter assay showed that miR-205 decreased the luciferase activity of E2F3 luciferase reporters obviously. (**G**–**I**) Expression of E2F3 in cells transfected with NC, miR-205 or miR-205 together with E2F3 (qRT-PCR, Western blot and immunofluorescence). (**J,K**) Glucose uptake assay was used to measure the glucose uptake of cells transfected with NC, miR-205 or miR-205 together with E2F3. (**L,M**) Migration assay was performed to explore the migration capacity of cells transfected with transfected with NC, miR-205 or miR-205 together with E2F3. (**N,O**) Transwell assay was applied to explore the invasion ability of cells transfected with NC, miR-205 or miR-205 together with E2F3. *P<0.05, **P<0.01. GAPDH used as control.

To explore the mechanism of miR-205 in glioma cell lines, we used starBase v3.0 to identify the potential targets of miR-205. Fortunately, we found that E2F3 may be a target of miR-205. Figure 5A shows miR-205 binding to the predicted sites of the 3′-UTR of E2F3. Next, we analysed the expression of E2F3 in glioma tissues and cell lines. The results showed that E2F3 was up-regulated in glioma tissues and cell lines compared with that in normal brain tissues and NHAs (Figure 5B–D). Then, luciferase reporter plasmids that contained wild-type and mutated E2F3 3′-UTR were constructed. Our data showed that the luciferase activity of the wild-type E2F3 3′-UTR vector was significantly decreased, while that of the mutant vector was not (Figure 5E,F). qRT-PCR, Western blotting and immunofluorescence demonstrated that the expression levels of endogenous E2F3 were down-regulated when miR-205 was increased and that the inhibitory effect of miR-205 on E2F3 could be restored by an E2F3 plasmid (Figure 5G–I). In addition, we also found that E2F3 restored the effects of miR-205 on glucose uptake, migration and invasion (Figure 5J–O). In summary, these results confirm that miR-205 plays a role through E2F3 in glioma cells.

### SNHG5 promotes the glucose uptake, migration and invasion of glioma cells by sponging miR-205 to up-regulate E2F3 expression

**Figure 6:**
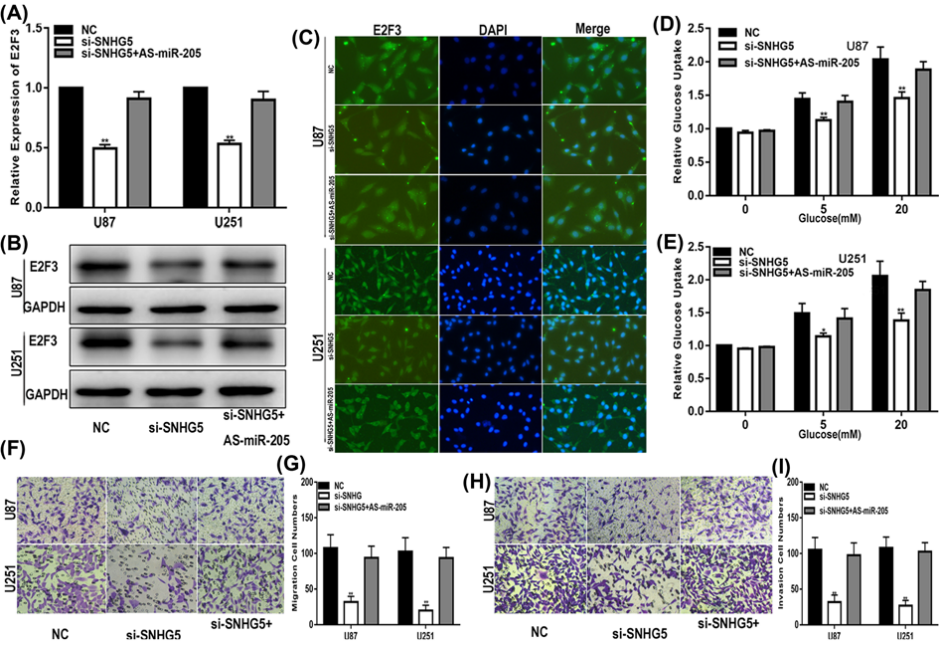
SNHG5 promotes the glucose uptake, migration and invasion of glioma cells by sponging miR-205 and up-regulating E2F3 expression (**A**–**C**) Expression of E2F3 in U87 and U251 cells transfected with NC, si-SNHG5 or si-SNHG5 together with miR-205 inhibitor (qRT-PCR, Western blot and immunofluorescence). (**D,E**) Glucose uptake assay was used to measure the glucose uptake of cells transfected with NC, si-SNHG5 or si-SNHG5 together with miR-205 inhibitor. (**F,G**) Migration assay was performed to explore the migration capacity of cells transfected with NC, si-SNHG5 or si-SNHG5 together with miR-205 inhibitor. (**H,I**) Transwell assay was applied to explore the invasion ability of cells transfected with NC, si-SNHG5 or si-SNHG5 together with miR-205 inhibitor. *P<0.05, **P<0.01. GAPDH used as control.

Our present study showed that SNHG5 promotes the expression of E2F3 in glioma. We intensively investigated whether SNHG5 drives the glucose uptake, migration and invasion of glioma cell lines via E2F3 in a miR-205-dependent manner. We transfected NC, si-SNHG5 or si-SNHG5 cells treated with the miR-205 inhibitor into glioma cells, and relative functional assays were designed and carried out. qRT-PCR, Western blotting and immunofluorescence were performed to detect E2F3 expression in glioma cell lines. The results suggested that decreasing SNHG5 down-regulated the expression of E2F3 and that the influence of si-SNHG5 on E2F3 was reversed by the miR-205 inhibitor (Figure 6A–C). Moreover, the effect of si-SNHG5 on the glucose uptake, migration and invasion of glioma cells was prevented by the miR-205 inhibitor (Figure 6D–I). Taken together, we found that SNHG5 modulates glioma progression by sponging miR-205 to up-regulate E2F3 expression.

### SNHG5 regulates tumourigenesis *in vivo*

**Figure 7:**
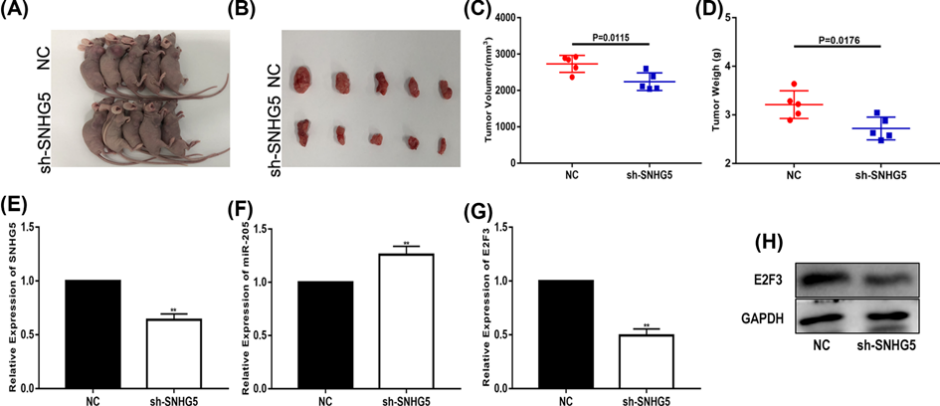
SNHG5 regulates tumourigenesis *in vivo* (**A,B**) Tumour formation in nude mice was achieved by injecting U87 cells. (**C**) Tumour volume from different groups. (**D**) Tumour weights from different groups. (**E**) SNHG5 expression was measured in tumour tissues by qRT-PCR. (**F**) MiR-205 expression was measured in tumour tissues by qRT-PCR. (**G,H**) E2F3 expression was measured in tumour tissues by qRT-PCR and Western blot. *P<0.05, **P<0.01. GAPDH used as control.

We constructed a glioma xenograft model to measure the role of SNHG5 *in vivo*. The tumours of U87 xenografts in nude mice are shown in Figure 7A,B. The results suggested that knockdown of SNHG5 efficaciously inhibited tumour growth. Compared with the control group, the volume and average weight of the tumours in the SNHG5 knockdown group were lower (Figure 7C,D). Additionally, the expression of SNHG5, miR-205 and E2F3 in the tumours was detected. The results verified that in the sh-SNHG5 group, the expression of SNHG5 and E2F3 was down-regulated and the expression of miR-205 was up-regulated (Figure 7E–H). These results demonstrate that SNHG5 could regulate glioma progression *in vivo*.

## Discussion

Recently, many studies have revealed the function of lncRNAs in various human tumours, which has increased our understanding of the molecular mechanism of tumourigenesis [21–23]. SNHG5, located in the introns of host genes, belongs to the category of non-coding genes and regulates gene expression by acting as a sponge for miRNAs. Although multiple studies have shown that SNHG5 plays an oncogenic role in some human cancers, including gastric adenocarcinoma, liver cancer and osteosarcoma [13,24,25], the mechanism of SNHG5 in glioma has not been thoroughly expounded. In our study, we reported that SNHG5 was overexpressed in glioma and found that SNHG5 promotes the glucose uptake, migration and invasion of glioma cells.

Numerous studies have revealed that specific lncRNAs can influence miRNA pathways through the ceRNA mechanism [12,20]. CeRNAs competitively bind to miRNA and inhibit miRNA binding to their targets, thus regulating gene expression. Once the balance between the ceRNA and the miRNA is broken, gene expression will be abnormal, thereby affecting tumourigenesis. For example, Wu et al. [26] showed that the lncRNA SNHG15 acts as a ceRNA to regulate the YAP1-Hippo signalling pathway by sponging miR-200a-3p in papillary thyroid carcinoma. Luan et al. [18] reported that the lncRNA H19 accelerates glucose metabolism and cell growth in melanoma through the miR-106a/E2F3 axis. In the present study, we hypothesized that SNHG5 might target miRNAs in glioma, and we identified the binding sites between miR-205 and SNHG5 through bioinformatics analysis. In addition, dual-luciferase reporter assays, RIP and RNA pull-down assays demonstrated that SNHG5 directly targets miR-205.

MiR-205 functions as a tumour suppressor in human cancers, such as breast cancer, prostatic carcinoma and oral squamous cell carcinoma [27–29]. Our study found that miR-205 suppressed the glucose uptake, migration and invasion of glioma cells. Then, starBase was used to determine the potential targets of miR-205. We identified that the 3′-UTR of E2F3 has binding sites for miR-205, and we found that E2F3 may be a functional target of miR-205. E2F3 belongs to the E2F transcription factor family, which can affect glioma progression [30]. Additionally, we verified that SNHG5 enhances the expression of E2F3 by competitively binding to miR-205 and that the effect of si-SNHG5 on glioma cells could be impaired by the miR-205 inhibitor. Finally, we demonstrated that SNHG5 enhanced the progression of glioma *in vivo*.

In summary, we revealed that SNHG5 is a novel oncogene in glioma. SNHG5 promotes the glucose uptake, migration and invasion of glioma cells by sponging miR-205 and up-regulating E2F3 expression. Our study not only furthers the understanding of the regulatory mechanism of the SNHG5/miR-205/E2F3 axis in glioma but also may lead to a new potential strategy for glioma therapy.

## Abbreviations

ceRNA: competing endogenous RNA
E2F: E2F transcription factor 3
GBM: glioblastoma multiforme
lncRNAs: long non-coding RNA
SNHG5: small nucleolar RNA host gene 5
